# Smart Device-Based Notifications to Promote Healthy Behavior Related to Childhood Obesity and Overweight

**DOI:** 10.3390/s18010271

**Published:** 2018-01-18

**Authors:** Gustavo López, Iván González, Elitania Jimenez-Garcia, Jesús Fontecha, Jose A. Brenes, Luis A. Guerrero, José Bravo

**Affiliations:** 1 Research Center for Communication and Information Technologies (CITIC), University of Costa Rica, San José 11501, Costa Rica; joseantonio.brenes@ucr.ac.cr (J.A.B.); luis.guerrero@ecci.ucr.ac.cr (L.A.G.); 2 MAmI Research Lab, University of Castilla-La Mancha, 13071 Ciudad Real, Spain; ivan.gdiaz@uclm.es (I.G.); jesus.fontecha@uclm.es (J.F.); jose.bravo@uclm.es (J.B.); 3 Faculty of Engineering, Architecture and Design, Autonomous University of Baja California, Ensenada 21100, Mexico; ejimenez@uabc.edu.mx

**Keywords:** ubiquitous computing, health, human-centered computing

## Abstract

Obesity is one of the most serious public health challenges of the 21st century and it is a threat to the life of people according to World Health Organization. In this scenario, family environment is important to establish healthy habits which help to reduce levels of obesity and control overweight in children. However, little efforts have been focused on helping parents to promote and have healthy lifestyles. In this paper, we present two smart device-based notification prototypes to promote healthy behavior with the aim of avoiding childhood overweight and obesity. The first prototype helps parents to follow a healthy snack routine, based on a nutritionist suggestion. Using a fridge magnet, parents receive graphical reminders of which snacks they and their children should consume. The second prototype provides a graphical reminder that prevents parents from forgetting the required equipment to practice sports. Prototypes were evaluated by nine nutritionists from three countries (Costa Rica, Mexico and Spain). Evaluations were based on anticipation of use and the ergonomics of human–system interaction according to the ISO 9241-210. Results show that the system is considered useful. Even though they might not be willing to use the system, they would recommend it to their patients. Based on the ISO 9241-210 the best ranked features were the system’s comprehensibility, the perceived effectiveness and clarity. The worst ranked features were the system’s suitability for learning and its discriminability.

## 1. Introduction

Obesity is one of the most serious public health problems of the 21st century and it is a threat to people’s lives [1]. Obesity is influenced by several factors. At a personal level, simple things such as eating alone or in the presence of other people can affect a person’s food intakes. Moreover, nutritional habits are also important factors influencing children obesity and overweight. For instance, restrictive eating practices are associated with weight gain [2]. Furthermore, people that insist on excessively control their eating behaviors show less ability to self-regulate food and energy intake across meals.

Not only human factors affect people’s behavior, there are also non-appropriate environments that impact on human beings at a social level. For instance, when people operate in contexts that offer larger than necessary food portions, that have a high availability of energy-dense foods, or strong influence for sedentary behaviors, living a healthy life becomes harder.

Several efforts have been conducted to prevent and delay the growth of this issue. Mobile devices, heart rate monitors, chest pins and other devices are used for lifestyle monitoring and self-control. Moreover, many interventions have been developed over the years to determine the proper way to promote healthy behaviors. Lau et al. described that when technological applications or devices are used for these purposes, the results are either as effective as other ways of intervention. The only exception is face to face interventions [3].

Many efforts have been put into using mobile technology to promote healthy behavior. External memory aids play an important role in supporting habit development; they are useful when they refer to the target behavior and the situation in which it needs to be executed. Even though the effectiveness and relevance of reminders decrease with time, reminders keep people engaged and help them to repeat the behavior, and in some cases, could support the start of the new habit, as the new behavior might develop faster than the decay of effectiveness of the reminder [4].

The effects of mobile-based notifications have been established in previous works. The amount of notifications received in a mobile phone is now unmanageable for users [5,6]. Therefore, we believe that combining mobile applications with pervasive computing through smart devices could have more impact in the people and enhance their user experience.

By smart devices we mean: instruments, equipment or machines that have their own computational capacity. These electronic devices are connected to a network and interact autonomously with other devices and users [7]. Moreover, smart devices also refer to devices that have properties of ubiquitous computing. 

In this paper, we describe a smart device-based system that helps to promote healthy behaviors with the aim of preventing overweight and obesity in children. Moreover, we present findings and suggestions for future developments obtained during the implementation and evaluation processes. The system has been applied in two scenarios; the first one intended to support parents in having a healthy snack routine, based on a nutritionist suggestion; the second scenario to deal with the problem of forgetting the required equipment on sports days, through notification reminders in the form of visual cues at proper timing. Both scenarios are focused on families with children between the ages 6 and 12, since it is important to take care of obesity in the early ages of the child as a strategy to promote healthy life, when physiology is more malleable [8]. Furthermore, the effects of the implemented actions are likely to be long-lasting. The goal of the system is to help people to establish routines that lead to healthier habits. Additionally, we want to use the proper technology and functionality to minimize user interaction and intrusion.

The system presented in this paper is developed in the context of a larger research with the goal of testing a conceptual framework to develop smart device-based notifications [9,10]. Moreover, the prototypes presented in this paper are developed using the knowledge gathered through the development of more than ten similar prototypes applied in four case studies. 

The system uses different technologies including low cost microcontrollers, sensors and simple actuators to deliver information to the users, a NoSQL database to model people and devices into the system, and a lightweight messaging protocol to allow the devices to work with low processing capabilities consuming small amounts of energy. 

The system was developed in the context of an international collaboration between Spain, Mexico and Costa Rica researchers. Moreover, the evaluation includes the perspectives of specialists in nutrition and physical activity from these three countries.

## 2. Related Work

Health and obesity problems related to physical activity and diet have been studied in the past. Moreover, some interventions have been conducted to assess the impact of reminders in these domains. This section presents related works to the one described in this paper. Even though there are many works focusing on comparing results of weight change, eating habits and physical activity in different contexts [11,12,13], we will only consider related works that use some kind of device or notification as a reminder for people.

In 2011, Monteiro et al., described a randomized controlled trial lifestyle intervention. Authors focused on nutrition and physical activity. Their study focused on mothers with young children (0–5 years old). The designed intervention used face to face workshops, emails and Short Message Service (SMS) reminders as notification mechanisms [14].

Also in 2011, Winett et al., described a program to assess nutrition, physical activity and body weight outcomes applying a social cognitive theory to health. This was a web-based intervention. Authors selected sedentary participants between 18 and 63 years old. With this intervention authors concluded that a simple web-based program can help people to comply with healthy behaviors [15].

Other studies focus more on behavior changing. For instance, in 2015, Hattar, Hagger and Pal, described a randomized control trial study protocol that developed psycho-education using implementation intentions and mental imagery (through videos). In this work authors developed and presented the protocol to evaluate the HEALTHI (Healthy Eating and Active LifesTyle Health Intervention) program. This is a theory-based intervention with the goal of changing dietary intake and physical activity behaviors in overweight people. Authors also propose the use of SMS in this work [16].

In 2016, Quintiliani and Whiteley, conducted a randomized trial to examine the feasibility of a nutrition and physical activity behavioral intervention. In this work, the communication mechanisms used were motivational phone calls from trained peer counselors. Even though authors reported no statistical differences between the control and intervention groups, the overall satisfaction with the program was high [17].

Finally, a category that has been study is the use of mobile apps to improve diet, physical activity and sedentary behavior. From studies combining SMS, emails, apps and websites [18], pregnant women using mobile phone as reminder and a Fitbit (Fitbit International Limited., San Francisco, CA, USA) as a control mechanism [19], textual and auditory cues delivered through a mobile app to increase fruit and vegetable intake and health literacy in general [20], all the way to mobile games using an activity trackers Tractivity (Kineteks Corp., Mainland, Vancouver, BC, Canada) as input to promote physical activity in children [21].

From the literature review conducted, we could not identify studies that used smart device-based notifications to promote healthy behaviors regarding snacking and physical activity to avoid or reduce overweight and obesity levels.

## 3. System Description

The user interface of our system consists of simple smart devices with embedded screens and visual LED cues that provide important information to the users in the right place at the right time. The idea behind these smart devices is that they are normal objects such as fridge magnets or cabinet drawer hooks that also have the capability of delivering information. The system has some key functions including: storing and processing data from the users, generating notifications and dispatching them to the properly smart device(s). 

The two prototypes described in this paper were designed and built following a conceptual framework for smart device-based notifications [9]. This framework proposes a modular conception of the system and defines guidelines for the decisions during the design process. The following sections describe the main components of the system.

Many of the design decisions of the two devices described in this paper are the result of lessons learned through the construction of several smart devices (included in the conceptual framework). One of the most important characteristics is the use of visual cues. Even though sound cues (auditory notifications) were strongly suggested by developers during the design process, the use of the framework lead to the decision of avoiding such notifications. The main reason to avoid sound notifications is that this type of notifications can easily become overwhelming. Moreover, visual recognition memory is superior to auditory recognition memory [22]. Furthermore, encoding messages in sounds is a difficult task; therefore, auditory notifications would have to present the full text of the notification to be effective.

### 3.1. Main Components

Our system is comprised of six main technological components. The first component, the physical activity tracker, is a device that family members use to gather their physical activity data and store it in the database. Activity bracelets attached to the wrist of children during the week provide information about steps, activity time, distance, calories burned, and quality of sleep among other features. Analysis of these data is later used to determine, for example, proper times of notification in conjunction with general information, food habits and anthropometric measurements that are manually collected. Recognition of parameters from activity bracelets can be performed in an automated and transparent. However, since we are using Xiaomi 1S (Xiaomi Inc., Haidian District, Beijing, China) smart wristband this process is semi-automated (i.e., data are synchronized with a smartphone via Bluetooth connection and requires a manual script). 

Physical activity data was collected using the activity band during a week. The data obtained was daily activity such as steps, activity time (hh:mm), distance (km), burned calories (cal) by day as well as the breakdown of all the activities carried out during the day. One week later the band was removed, the data was synchronized to the Band App and finally we used a script to transfer the data to our database.

All the gathered data are stored in a central database (2nd component). In this project, we have used a non-structured document database built using MongoDB v3.6. The purpose of this is to take advantage of the flexibility and scalability provided by this technology. Another reason to build this system using MongoDB has been the necessity of adaptability during the development and evaluation of the solution. Depending on the user’s characteristics, the available data differs. Therefore, a traditional structured approach is not appropriate.

The third technological component is the notification generator. The conceptual framework applied in the development of this system was conceived to be applied in several contexts. The name notification generator specifies a piece of software that uses the gathered information and environment conditions to generate notifications. However, these notifications do not have to be automatically generated. In the case of our system the notifications that are delivered through the prototypes are generated based on the recommendations of specialists in nutrition and physical activity (i.e., the information and parameters for delivery of each notification are predefined). However, the system could be used to generate automatic notifications based predefined conditions and the information gathered and stored in the central database. 

The notification generator, in our system, is a piece of software developed to use the predefined information stored in the central database to create a notification. Moreover, this part of the system includes the parameters for delivery, also predefined, to establish the delivery interval of each notification in accordance with the timing specified by the specialists. Generated notifications are stored in the central database, because the notification dispatcher extracts notifications from this centralized repository to be delivered. 

For the specific purposes of the implementations of the framework described in this paper, the notifications are listed and specified by specialists in nutrition and physical activity. This list of notifications is stored in the database and specific parameters are established to determine when the reminder should be delivered. Moreover, all the available information (stored in the database) is used to determine if the notification can be delivered through the predefined device (i.e., fridge magnet, cabinet drawer or other available devices). An example of the use of this information is the use of sleep patterns to determine an appropriate time to display notifications. The use of a non-structured document database allows different types of data to be stored and used in whichever way the notification requires. A more detailed explanation of the notifications delivered is described in Section 4 (Application Domains).

The fourth component is the notification dispatcher. This is a software component in charge of serving notifications to their final recipient devices. The notification dispatcher functions as a link between the system and each dedicated device. This approach is required to address the problem of standardization in the smart device domain. Each smart device offers different functionalities; therefore, different ways to deliver notifications must be implemented.

The notification dispatcher allows communication between the database and the smart notification devices through the Message Queuing Telemetry Transport (MQTT) protocol. MQTT is a publish-subscribe-based lightweight messaging protocol that runs on top of the TCP/IP protocol. Publish-subscribe is a communication pattern where senders of messages, called publishers (e.g., notification dispatcher), do not route messages to be sent directly to subscribers (e.g., notification devices), but instead characterize messages into topics and publish/post them on a broker. The subscribers then receive the messages from the broker depending on the settings of their connection and the topics they are subscribed on. The broker (5th component) consists of a dedicated server placed between publishers and subscribers to manage message dispatching and to ensure delivery through different quality of service (QoS) strategies that reinforce the delivery mechanisms of the TCP/IP protocol. 

The use of a MQTT broker between the notification dispatcher and the notification devices, instead of a direct link, was a deliberate decision. QoS level 1 and QoS level 2 policies ensure that messages (notifications in this case) reach the target device/s. If a target (notification) device is unreachable at a time, for instance, due to an accidental disconnection or because it has no power, the MQTT broker will store undelivered notifications and will serve them, when the device is available again. In addition, advanced QoS strategies play an important role in energy saving enabling the use of power save configurations in the notification devices. Thus, the duty cycle percentage, which is power demanding, may be substantially reduced in the notification devices by entering deep sleep mode periodically. If there are notifications attempts while target devices are sleeping, they will be forwarded by the MQTT broker and received in the upcoming duty cycles.

Finally, the last components are the smart devices used for notification delivery. In the present work, two dedicated devices have been created from scratch. Currently, there are several smart devices available for purchase. However, these devices do not provide the proper functionalities to deliver information and gather responses from users as it is intended with our system. A review of available IoT devices was conducted before deciding to implement the two smart devices described in this paper. Even though we found more than 85 smart devices (with different functionalities) none was sufficiently customizable or provided all the functionalities that the developed prototypes offer. Appendix A shows the list of smart devices studied before deciding to develop the dedicated prototypes. 

The two prototypes of notification devices that have been built in the present work are equipped with a low-cost System on a Chip ESP8266-12E (Espressif Systems Inc., Shanghai, China), which integrates a 32-bit RISC Tensilica Xtensa LX106 micro-controller and an 802.11 B/G/N Wi-Fi transceiver to support wireless communication and Internet access through the TCP/IP protocol stack built in the firmware. MQTT client code supporting publications and subscriptions to topics with QoS level 1 has been implemented and compiled to run on top of the TCP/IP protocol. In addition, the ESP8266-12E enables Inter-Integrated Circuit and SPI (Serial Peripheral Interface) serial communications to connect different sets of sensors and actuators.

Both prototypes have been specifically designed to deliver notifications that promote healthy behaviors in children. The most complex consists of a fridge magnet that includes a 0.96-inch monochrome OLED graphic display, which has been connected through the SPI bus to the micro-controller. It has 128 × 64 pixels that can be used to display static or dynamic monochromatic images and text messages. Furthermore, this prototype is equipped with a single InvenSense MPU-6050 6-DOF IMU (InvenSense Inc., San Jose, CA, USA) wired through the I2C interface. It has been included to support feedback actions in notifications, such as “opened/closed fridge door alarm”, among others. This prototype is focused on delivering notifications that promote healthy eating behaviors through the fridge door, as it is a right place to do it. This decision is since kitchen surfaces, particularly fridge doors, are frequently used as reminder systems at home-specific locations [23]. Shopping lists, to-do lists, tailored messages, and paper reminders are often left on the fridge. In this context, emphasis is placed on the importance of fridge magnets as they are seen to contribute to a fluidity and configurability that make fridge surfaces unique to provide contextual cues to the users [24]. Therefore, fridge doors are appropriate places to deliver proactively notifications that support habit formation and behavior changes [25]. Specifically, the magnet prototype is well-suited to provide notifications in the form of visual cues which persuade users to prepare snacks in advance promoting healthy eating. The hardware scheme of this first prototype can be seen in Appendix B.

On the other hand, the second prototype for notification delivery focuses on promoting physical activity in children, another factor to reduce overweight and obesity levels. It has no display and it relies on a simplest resource for providing notification awareness. In this case, the prototype consists of a five RGB LED array with integrated drivers from Adafruit (Adafruit Industries Inc., New York City, NY, USA). A relevant keyword (“Sports Day”) is carved out on the prototype 3D printed plastic case, so that it allows the light to pass through the letter gaps highlighting the keyword. Furthermore, this prototype is equipped with a push button not to turn the notification LED lights off, but to give feedback to the system so that the notification has been attended by the end users. 

**Figure 1:**
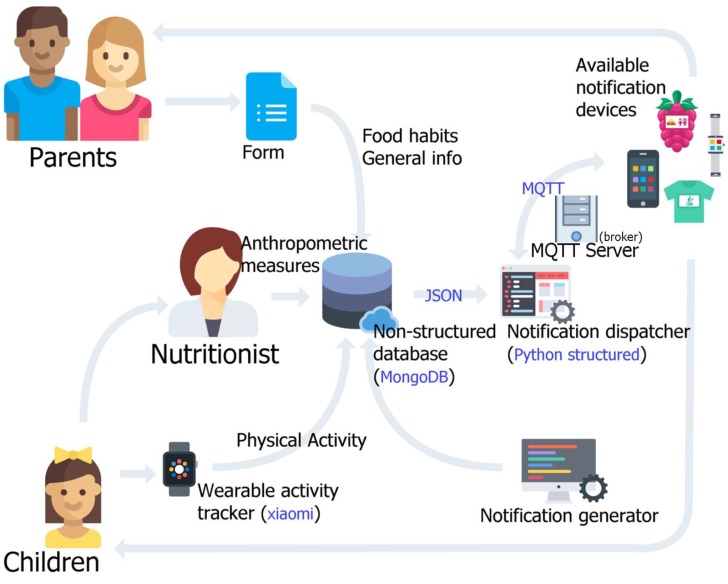
System overview including: main actors, software components, and technological infrastructure.

It has been designed in the form of a cabinet drawer hook, as it is a right place for sportswear reminders. This assertion is supported by two basic arguments. The first is that the cabinet is de facto a usual place to store sportswear so that visual cues on sports days, at proper timing, ensure that the notification has been seen. The second reason is purely technical; since the prototype incorporates a built-in Wi-Fi transceiver intended for its use at home settings. The prototype is designed to remain static in the context of the household. An overview of the system including main actors, software components and technological infrastructure is depicted in Figure 1.

To the best of our knowledge, there are no further studies that attempts to persuade users to not forget their sportswear promoting physical activity through notification reminders in this context. In 2015 Lau, Wong, Luk and Kwok [26], described the problem of forgetting important items depending on the daily school schedule. It was addressed through a reminder system based on near field communication. Particularly, this system was integrated into a school bag and it provided reminders about items such as lunch boxes and sportswear, among others. Nevertheless, the prototype in [26] is considered for mobility beyond the household.

The hardware scheme of our second prototype focuses on promoting physical activity in children through sportswear notification reminders can be seen in Appendix C.

Both prototypes use 3.7 volts lithium polymer batteries and they are complemented by a regulator circuit to provide stable 3.3 volts to the different modules, together with a charge controller that regulates the charge process and avoids the complete discharge of the battery during use. Moreover, both prototypes have 3D printed cases. Table 1 overviews detailed specifications of these two prototypes.

### 3.2. Modeling the Notification Message Structure

As stated in the Main Components section, the notification generator can build customized notifications and plan their delivery by using the information stored in the non-structured database. Notifications are dispatched to the proper smart devices thanks to the notification dispatcher and the MQTT broker. In addition, the recipient devices also have the technical ability to send acknowledgement notifications to the dispatcher (if required).

To build a flexible and scalable system, the notification message structure has been modeled to be independent of the notification mechanism, the application’s context and the target device.

Notifications are encapsulated within MQTT messages and therefore, both, the notification devices and the notification dispatcher should implement the MQTT stack and operate as MQTT clients. While the notification devices use an embedded lightweight version of the MQTT client which can be loaded into the memory of the microcontroller; the notification dispatcher, for its part, implements a full-featured standalone client.

Since the non-structured database and the logic that governs the notification generator and the notification dispatcher work with JSON data, the notification has also been modeled as a JSON object which can be enclosed in the MQTT message, instead of using plain text. The JSON syntax provides a flexible mechanism to build a general notification scheme that can be adapted to many application’s contexts and notification devices.

The use of this notification model is very valuable. When the notification is defined and queued to be delivered, the generator does not know which device would be used to deliver the notifications. Therefore, a flexible format allows storing the notification content independently to other metadata. Moreover, the same notification can have content in different formats (e.g., audio, text, and figure) and that information would be used depending on the device that is selected to deliver the notification.

## 4. Application Domains

The primary requirement pursued in the developed system has been to make it easy to use and accessible. Therefore, the two prototypes have been designed to interact with the user through very simple visual cues as mechanisms to address notification delivery regarding promotion of healthy life in terms of reducing obesity and overweight in children population. By helping with eating habits, the first prototype promotes a healthy snacking behavior; by implementing a visual reminder for sportswear, the second prototype prevents self-sabotage or simply forgetting the required equipment to practice physical activity when it is sport day.

### 4.1. Healthy Snacking Behavior

Meal frequency and timing patterns are two of the most important factors that affect obesity rates [27]. Snacking is an important part of the eating habits around the world. A snack could consist of chips, nuts, cheese, yogurt, cookies or biscuits, vegetables, fresh fruit, chocolate or other foods. Snacks are supposed to be an in-between meals food. The 2014 Nielsen global snacking report [28] stated that nutrition is the most important reason to eat snacks, followed by getting an energy boost. Therefore, healthy snacks are important.

There are two types of snackers. The first are planners, those who prepare or purchase their snacks in advance. These types of snackers are usually very selective. Second, the spontaneous snackers. These snackers often eat snacks as soon as they buy or prepare them without previously planning. The latest usually lack a regular eating pattern, not considering the health and nutritional aspect of the snack.

Getting healthy snacks in a spontaneous purchase without a steady provider is a difficult task. Normally, when you buy snacks on the street they are not healthy. In this context, the purpose of our system is to provide a reminder for snack planners and for spontaneous snackers who want to plan their meals and adopt regular snack eating patterns, taking care of nutritional and healthy aspects and making this behavior extendable to the entire family unit.

**Figure 2:**
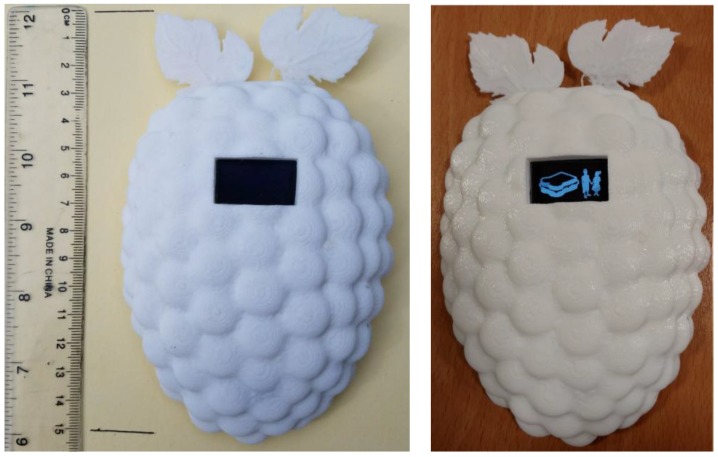
Prototype developed to promote healthy snack behavior and one example of notification delivered. (**Left**) Prototype powered-off and case, ruler for reference; (**Right**) Prototype powered-on, displaying a sandwich notification for two children.

Our prototype is a snack reminder developed to deliver personalized notifications to support parents in remembering snack preparation and reminding them to deliver the snacks to their children before they leave the house. As a fridge magnet, this device is supposed to be an ornament. However, the embedded hardware makes it capable of delivering graphical notifications to anyone with access to the fridge. Figure 2 shows the prototype and the aspect of one of the notifications delivered.

The system works based on the recommendations of a nutritionist. Either through a generic snack diet or a personalized diet depending on the circumstances. Given a diet proposal, the system sends a list of required ingredients for the week snacks. Then, graphical cues to remind parents which snacks they are supposed to give their children are daily delivered.

The notification delivery mechanisms could also be used to send visual cues as reminders the day before if parents require to prepare the snack in advance.

The way in which these notifications are defined is justified by the fact that for overweight children, programs that involve the parents and the home setting are better to promote healthy eating habits. Moreover, presumably the childhood period has a strong influence on the weight through life [29]. To provide some of the rationale for these notifications, we used The Five W’s, shown in Table 2.

To summarize, the healthy snacking behavior prototype provides notification based on recommendations of specialists in nutrition that create a list of snacks per family member (specially focused on children). These notifications are delivered through a smart device (fridge magnet) during the morning. The intention of the notification is to reinforce parents desire to prepare healthy snacks and to avoid forgetting to pack the snacks for their children. The snack recommendations are stored through simple web form interface.

To deliver the notifications all the information stored in the database is considered and a set of rules and scripts define the time slots in which each notification should be delivered. The system also includes a feedback mechanism that is activated when someone opens and closes the fridge door.

### 4.2. Physical Activity (Sportswear)

**Figure 3:**
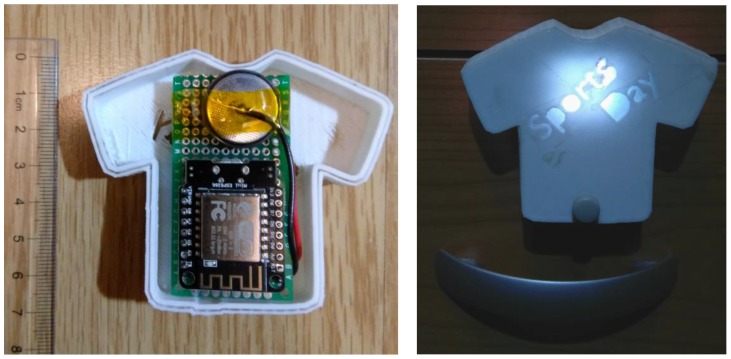
Prototype developed to remind people of sportswear to promote physical activity. (**Left**) Dedicated device and case, ruler for reference; (**Right**) Device powered-on.

There are always those days in which people intend to do something, but they search for any excuse to avoid it. For physical activity, one of those is to forget sportswear. Getting to do sports with your children or going to the gym requires preparation and habits if people want it to become part of their routine. Figure 3 shows the prototype.

Willpower is also a limited resource [30]. If people continuously forget something necessary for their physical activity, a change is required to avoid it. People could also use little reminders (for example post-its), however, this will require them to place the post-its somewhere. Furthermore, post-its provide static information; this means that they do not dynamically adapt the information contained to the actual context (in this case to the current day of the week). That is why this system uses visual cues to remind a person that today is a day for physical activity or not.

Using a small dedicated device that can be hooked, for example, in a cabinet drawer our system delivers a notification that help users remind carrying sportswear. This may also be combined with the snack notification device to remind users to carry water bottles or other required snacks for hydration and energy recovery. Table 3 shows the rationale for this notification.

## 5. Evaluation

The evaluation was conducted through a series of interviews with specialists in nutrition and physical activity from three different countries: Spain (one male and two females), Mexico (two males and one female) and Costa Rica (one male and two females).

For each interview, a physical prototype and a storyboard were presented to the participant and a series of questions were asked. In each session, the same interview protocol was used. The protocol consisted of three main parts: participants’ information, prototype usefulness and prototype usability/ergonomics. The questions in the protocol are detailed in Appendix D.

The goal with this evaluation was to assess the system’s perceived usefulness and usability. Moreover, the thoughts of the specialists were gathered and added to the prototypes.

The system was presented using the physical device and storyboards [31]. Using storyboards, we presented the main use cases for the prototypes and their benefits. Participants were presented with two storyboards, one for healthy eating behaviors and the other for sportswear reminders.

The developed interview protocol and questionnaire was designed using two main references: The first set of questions (focused on the prototype) were based on different scales [32] with the intention of gather the perspective of the participants on the usefulness of the system. The second part was based on the standard ISO 9241-210:2010 Ergonomics of human-system interaction [33].

To provide a better understanding we will describe our interpretation of the ISO 9241-210:2010 terminology. By effectiveness we mean the accuracy and completeness with which users achieve specified goals. By efficiency we mean resources expended in relation to the accuracy and completeness with which users achieve goals. By satisfaction we mean freedom from discomfort and positive attitudes towards the use of the product.

Conciseness, consistency and self-descriptiveness deal with the amount of information required to deliver the message and the way in which this data is displayed every time. The characteristics of presented information (i.e., detectability, legibility, discriminability, clarity and comprehensibility) are related to freedom from distraction and interpretability. 

Finally, suitable for individualization means that, users can modify interaction and presentation of information to suit their individual needs and suitable for learning means that the system conforms to user’s expectations and it prevents or tolerates errors.

## 6. Results and Discussion

**Figure 4:**
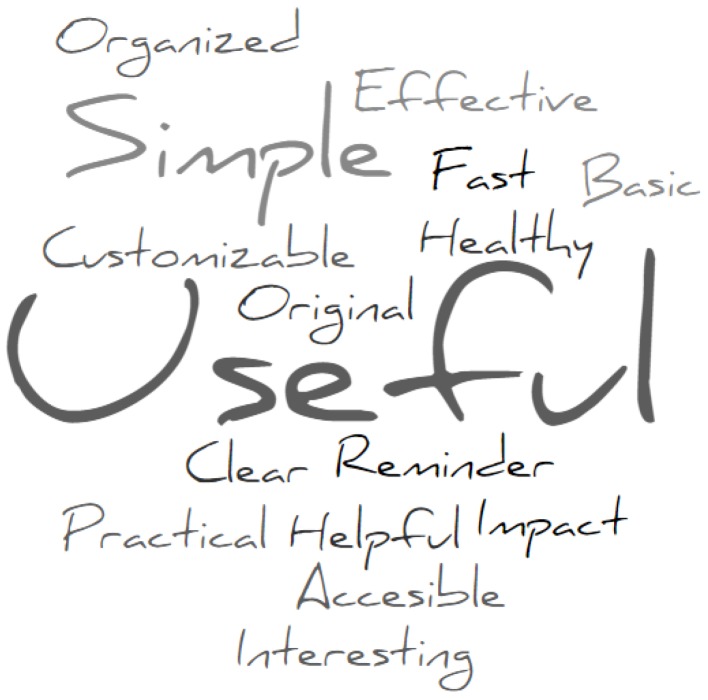
Word cloud of main characterizers used by evaluators.

In this section, we present the results of the interviews. The evaluation protocol was explained in the Evaluation Section. All results are positive. However, some of the evaluators provided feedback and improvement opportunities that we will also discuss. We asked the evaluators to use three words to describe the system. Figure 4 shows the results of this exercise. The most common word used to describe the system was useful (5 out of 9) followed by simple (4 out of 9).

Some words caught the interviewer’s attention. For instance, when a reviewer used the word “accessible”, we followed up to determine that she was referring to the possibility of many people to reach the information on the device. This includes everyone in the household and since the system uses graphical representations it is also accessible for children that do not know how to read or write. 

One of the interviewees also used the word “basic” we followed up to determine that she saw potential to add other functionalities to the device. Therefore, the version presented was too basic. Another word was “organized” delving in this we determined that the characterization was for the users and not the system. Therefore, organized people are potential users of the system.

**Figure 5:**
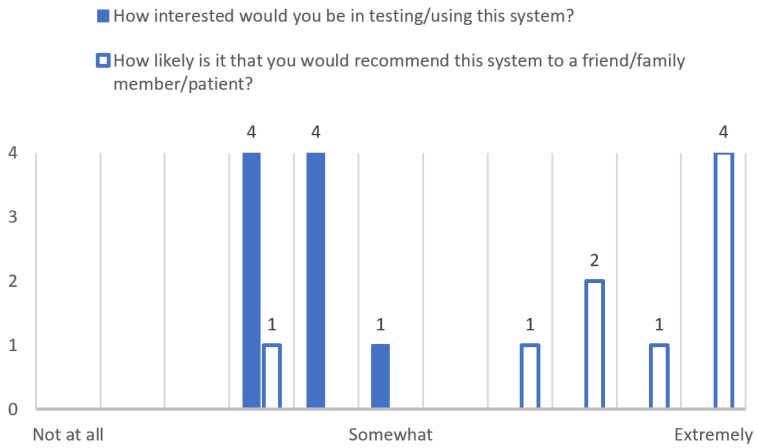
Likelihood to use the system or recommend the system.

Two questions were asked to the evaluators to assess their willingness to either use the system or to recommend it to their friends, family or patients. Figure 5 shows the answers to this question. It is interesting to observe that nine out of nine evaluators (specialists in nutrition and physical activity) are somewhat interested in using the system. However, eight out of nine evaluators consider that they would recommend the system. To further explain this, we must note that usually specialists in nutrition and physical activity have a healthy lifestyle, therefore they might not see fit to use the system. However, during the interviews most of the evaluators stated that remembering healthy snacks was one of the main problems of their patients.

**Figure 6:**
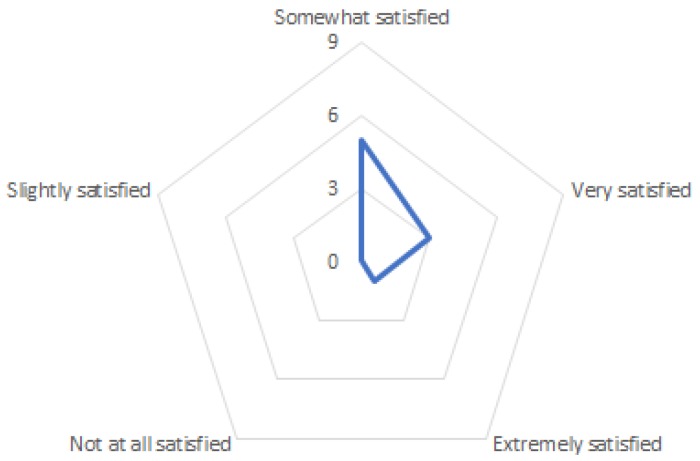
Evaluator’s satisfaction with the look and feel of the system. Scale (0, 3, 6, 0) represents the number of participants in each category.

Some of the evaluators stated that they would not use the system as it is. But they would reconsider if it included some of their feedback (especially screen size and the amount of information that can be delivered through the device). The evaluation had two main focuses, the first one was to assess the evaluator’s opinion on the system, the second one was focused on the look and feel of the device (human factors and ergonomics perspective). We asked a question regarding the evaluators’ satisfaction with the look and feel of the devices. All the answers were positive (5 somewhat satisfied, 3 very satisfied and 1 extremely satisfied). Figure 6 shows the responses to this question.

Most of the improvement opportunities mentioned by the evaluators include: enlarging the device screen and making it in color, changing the shape of the case, adding sound to the notifications, and adding text to the notification and not only images. This were the main concerns regarding the device.

One of the evaluators recommended to add text, this text would be used to provide information about the ingredients of the snack. In Costa Rica, the two of the evaluators asked to change the shape of the snack reminder case to an apple as it is traditional in Costa Rica to have an apple shaped magnet fridge.

Another important observation was that some of the evaluators compared the system with mobile applications with similar functionalities. Other evaluator requested for the system to sync with mobile applications.

Delving on the device functionalities, most of the comments were focused on the snacking reminder device. The first recommendation was to add reminders not only to people to carry their snacks but also to prepare them in advance. This would be helpful to assure that the snacks are ready to go when they are required. One evaluator recommended to use only one device with more information (i.e., combining both devices).

**Figure 7:**
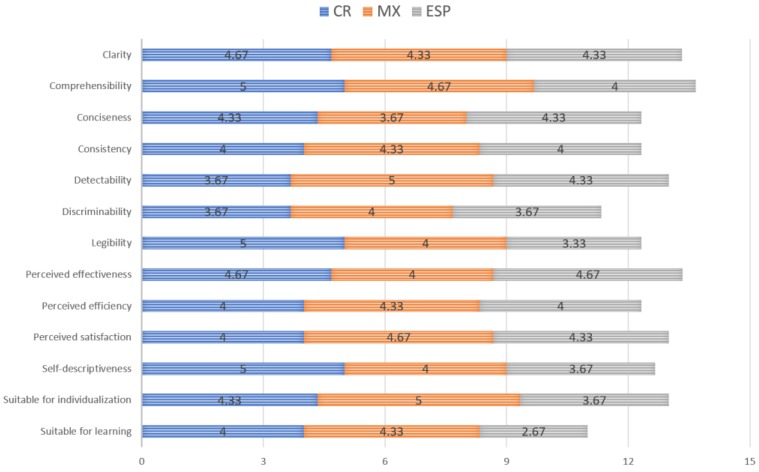
Evaluation of system characteristics based on the standard ISO 9241-210:2010.

The last part of the evaluation focused on the ergonomics of human-system interaction. In this section, 13 characteristics of the system were assessed by the evaluators. Figure 7 shows the results of this evaluation segmented by country. Table 4 shows the five-number summary and a reliability analysis of the data depicted in Figure 7.

An interesting finding of this evaluation is that both Mexico and Costa Rica on average consider the system good (4.3 out of 5 points). However, in Spain, the opinions were less positive (3.9 out of 5 points). We do believe that being Latin-American countries, Costa Rica and Mexico share opinions on how the system could be used.

In general, the best ranked categories were system’s comprehensibility, the perceived effectiveness and clarity (4.5 out of 5 points). The worst ranked categories are the system’s suitability for learning and it discriminability (3.7 out of 5 points).

A system is suitable for learning when it supports and guides the user in learning to use the system. In our case since the notification and device are supposed to share little information the suitability for learning suffers. To be able to learn to use the system a simple guide should be provided. The discriminability problem is mostly related to the device screen size and resolution. The implemented prototype used a 0.96-inch monochrome OLED graphic display; to improve this, a bigger screen should be used. Moreover, images that avoid ambiguity could be used. Enlarging the screen would also allow for text to be shown, improving discriminability of the notifications.

The final improvement opportunities for the system are focused on the notifications. One evaluator suggested adding personalization based on the user’s data and preferences (already considered in the system). However, she added the possibility to add diseases and allergies into the notifications. Another evaluator suggested the necessity of creating profiles (also considered in the system), but he requested to be able to change between profiles using the device.

In general, one recommendation is to add more nutrition facts of the snacks. For instance, alimentary groups, portion sizes and water consumption.

One confronted opinion between evaluators was if the personalization should be performed by the user or if the definition of snacks should be personalized by the nutritionist. One of the evaluators suggested that she would like to be able to specifically set the food characteristics for a given day in their patient’s device. Another evaluator suggested doing a consumption analysis of the user. However, this would require sensing capabilities that the system does not provide as it is designed for notifications.

The last opinion on the system is that two evaluators would prefer this to be a mobile application rather than a physical device. The main reason provided is that most people already have a mobile device. During the design phase of this system, this discussion was carried. 

One of the main arguments to avoid push notifications on smart devices is that the indiscriminate use of these notifications is causing people to stop paying attention to them. Social media notifications, dedicated apps (e.g., Netflix, Yelp, and Amazon), email, and other mobile phone notifications are just some examples of this overwhelming amount of notifications.

Finally, all evaluators highlighted the positive effect of using these devices in day to day to prevent overweight and obesity if activities and tasks associated with notifications are carried out properly.

## 7. Conclusions and Future Work

In this work, we presented the design, implementation and expert evaluation of two smart devices used to promote healthy behaviors (i.e., healthy snacking and physical activity). We presented the technical details of the device implementation and software architecture based on a framework to develop smart device-based notifications.

The prototypes were evaluated by nine specialists in nutrition and physical activity from three countries (i.e., Spain, Mexico and Costa Rica). Results show that the system is well received by the experts, and that they will be willing to recommend the system to their friends, family or patients.

The system was evaluated applying a standard categorization for ergonomics in human-system interaction. The best ranked categories for this evaluation were comprehensibility, effectiveness and clarity. This leads us to think that the system will be usable, and it will provide a nice user experience. The worst ranked categories were suitability for learning (i.e., the system does not teach the user how to use it) and discriminability. The discriminability problem is mainly due to the use of a small graphic display in the prototype.

Furthermore, we observed that the degree of satisfaction in Mexico and Costa Rica are the same. However, evaluators from Spain are more reluctant about the system. According to the expert evaluators (eight out of nine), the system proposed in this paper would be useful to promote healthier behaviors in the system users. The only negative response believes that providing the notification is not sufficient, but also more control should be provided by the system.

In future work, we will improve the system based on the expert suggestions and evaluate it with families in their households, integrating the analysis of physical activity parameters to support the enrichment of adapted notifications provided by the system. 

Furthermore, we will investigate how human activities are affected by this kind of smart device based notifications. Also, if users make responsible and proper use to achieve what smart devices pretend by adding new functionalities regarding persuasion and motivation focused on other domains of application, not only childhood obesity and overweight.

Although this research was prepared and executed carefully, it still has some limitations. First, the evaluation sample size (nine specialists in nutrition and physical activity) is reduced. This is due to the difficulty to personally work with specialists. Researchers considered applying an online survey to reach a larger sample; however, the final decision was to conduct personal interviews to focus the evaluation in the prototypes usefulness and its usability/ergonomics. Another limitation is that the evaluators are from different countries. Therefore, they might have different perspectives and points of view. However, the selection of three Spanish-speaking countries with cultural similitudes was a key factor to conduct the research as it is described in this paper.

## Figures and Tables

**Table 1:** Dedicated devices hardware characterization.

Characteristic	Healthy Eating Behaviors	Physical Activity Reminder
Communications	802.11 B/G/N Wi-Fi connection with Internet access/Lightweight client implementation of the MQTT protocol with QoS level 1 publish/subscribe services	802.11 B/G/N Wi-Fi connection with Internet access/Lightweight client implementation of the MQTT protocol with QoS level 1 publish/subscribe services
Processor Power	32-bit RISC micro-controller, 80 MHz CPU clock speed, light/deep sleep power modes	32-bit RISC micro-controller, 80 MHz CPU clock speed, light/deep sleep power modes
Memory	160 KB RAM, 4 MB external SPI flash	160 KB RAM, 4 MB external SPI flash
Operating Voltage	3.0–3.6 V (3.3 V LDO voltage regulator)	3.0–3.6 V (3.3 V LDO voltage regulator)
Power Consumption	Reduced time in deep sleep mode (interrupted by MPU-6050 IMU readings to sense fridge door)	Longer time in deep sleep mode (waking by a 1 to 10-min timer is enough)
	Deep sleep power < 10 μA	Deep sleep power < 10 μA
	During transmission in duty cycle ~120–140 mA	During transmission periods in duty cycle ~120–140 mA
Functionality	Show graphical notifications in a monochrome OLED screen/Sense fridge door	RGB LED array ON and OFF actions/Push button to indicate notification awareness
Cost	$35	$30

**Table 2:** Five Ws description of snacking behavior notifications.

Five Ws	Description
Who will receive notifications	The snack notifications are directed to parents who want to either prepare healthy snacks or avoid forgetting to give the snacks to their children.
What will be notified	In the case of preparation notifications, parents will be reminded of the type of snack their children are supposed to take to school. The other notifications help parents to avoid forgetting to provide the snacks, therefore they show which snack corresponds to each family member.
When will the notifications be delivered	These notifications are delivered early in the morning or one day in advance to help people remind that they should prepare snacks. The notifications are also present when someone is supposed to leave the house depending on their daily routine.
Where will the notifications be displayed	The smart device for these notifications should be placed in the fridge. As it is a fridge magnet. This allows people to associate the notifications with food and seemingly forces them to act immediately as they are already in the right place to prepare or pack their snacks.
Why will the notifications be delivered	These notifications help people who usually do not prepare their snacks to do it. Also, they avoid that parents forget to pack their snacks. This leads to a healthier eating behavior.

**Table 3:** Five Ws description of sportswear notification.

Five Ws	Description
Who will receive the notification	In this case the notification could either be directed to the children or the parents.
What will be notified	This notification will only show an illuminated case with the word “Sports Day”. The idea is that this notification will provide enough information to remember that it is a sports day.
When will the notification be delivered	This notification will be delivered on days that are planned to be sports days. This is decided by the parents and configured in the system (notification generator).
Where will the notification be displayed	The device will be placed in a closet drawer of either the parents or the children.
Why will the notification be delivered	Forgetting sportswear is one of the most common excuses to avoid physical activity [30]. Therefore, having this constant reminder on sports day would make people less likely to forget unconsciously.

**Table 4:** Minimum, maximum, quartiles and reliability analysis for Likert scales depicted in Figure 7. All items Cronbach Alpha: 0.8307, Std. Alpha: 0.8431. Reliability analysis calculated using [34].

Characteristic	Min	1st Quartile	2nd Quartile	3rd Quartile	Max	Cronbach Alpha
Clarity	4	4	4	5	5	0.8350
Comprehensibility	3	4	5	5	5	0.8106
Conciseness	3	4	4	4	5	0.8157
Consistency	3	4	4	4	5	0.8139
Detectability	3	4	4	5	5	0.8425
Discriminability	3	3	4	4	5	0.8186
Legibility	2	3	5	5	5	0.8056
Perceived effectiveness	3	4	5	5	5	0.8391
Perceived efficiency	3	4	4	4	5	0.7919
Perceived satisfaction	4	4	4	5	5	0.7981
Self-descriptiveness	3	3	5	5	5	0.8051
Suitable for individualization	3	4	4	5	5	0.8412
Suitable for learning	1	4	4	4	5	0.8135

**Figure A1:**
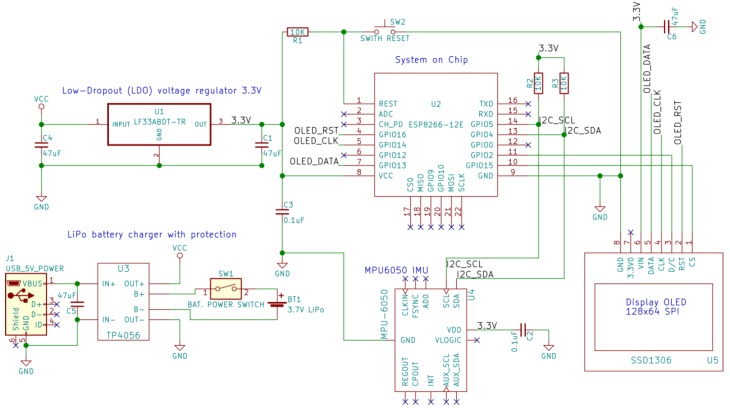
Hardware scheme of the smart device for healthy eating behaviors.

**Figure A2:**
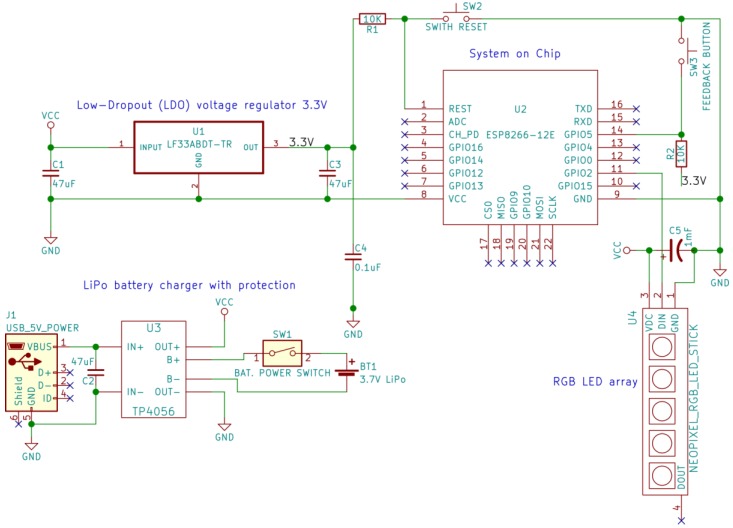
Hardware scheme of the smart device for promoting physical activity.

## Appendix A

This appendix lists the smart devices considered before deciding to develop from scratch two dedicated devices.

**Table A1:** List of smart devices revised durin the development of this research.

Smart Devices		
Kolibree	Breeze: Wireless breathalyzer	CubeSensors
iBGStar Blood Glucose Meter	TP-Link: Multicolor smart wi-fi	AwairGlow
CINDER: Sensing cooker	Mimo: Smart baby monitoring	Wemo Mini: Wifi smart plug
Vessyl Cup	UP3: Health tracker	Honeywell Lyric T5
Tagg Plus: Pet tracker collar	Ring: Video doorbell pro	SmartMat: Intelligent yoga mat
Cue	Misfit Wearables: Shine 2	Nest Cam: Outdoor
HAPIfork	June: Intelligent oven	Foobot: Indoor air quality monitor
TAO	Apple TV: Apps and voice control	August: Smart Keypad
Keen Home: Smart vent	Philips Hue Bridge: Homekit ready	Philips Hue: Dimmer switch
Fitbit Surge: Fitness tracker	Osmo: Gaming system for iPad	Samsung Family Hub Refrigerator
Flip 2: Wearable for kids	Deeper: Smart portable fish finder	Bluesmart: Connected Carry-On
Withings: Wireless blood pressure monitor	Garmin Vivosmart: Fitness band and smartwatch	Luna: Turn your bed into a smartbed
Talkies: A new way to chat with kids	iBaby Monitor: Digital video monitor with night vision	ShutterEaze: Automate your plantation shutters
FitBark: Activity tracking for your pet	Olive: Intelligent bracelet that helps you manage	Google Home: Speaker with Google Assistant
Google Daydream: Virtual reality headset	Samsung Galaxy View: Connected screen	Logitech Pop: Smart button controller
Logi Circle: Portable Wi-Fi video camera	Onyx: Wearable communication device	Ray Super Remote: Touchscreen universal
AWS IoT Button: Programmable dash button	MOTA SmartRing: Connectivity at your	CUJO: Smart firewall for the smart home
MUZO Cobblestone: Wireless music receiver	Aether Cone: The thinking music player	Xiaomi Yeelight: Indoor connected night light
Awair: Smart air quality monitor	August: Smart lock	iRobot Mirra: Pool cleaning robot
Refuel: Smart propane tank gauge	Kinsa: Smart thermometer	Roku 3: Streaming media player
Belkin WeMo: Insight Switch	Click & Grow: Smart herb garden	Amazon Echo Dot: 2nd Generation
Amazon Tap: Portable voice controlled speaker	Prodigio: Connected Nespresso machine	Solu: Smallest general-purpose computer
Google Chromecast: Second generation	Amazon Dash Button: Smart home shopping	Philips Hue Go: Portable connected lighting
Singlecue: Gesture control for connected home	TAH: Bluetooth device for Internet of things	Valta: Simple home energy management
Netatmo Welcome: Smart camera with face	Lutron Caseta: HomeKit-enabled smart lighting kit	Sengled Pulse: LED light + wireless speakers
Roost: Smart battery for smoke alarms	Point: A softer take on home security	Scout Alarm: Simple connected home security
LIFX: White 800 connected light bulb	Ambi Climate: Smart add-on for your air conditioner	Allure EverSense: Proximity based thermostat
Rico: Turn your smartphone into a smarthome device	Highfive: Video conferencing you can actually love	Smappee: Smart home energy manager
Chamberlain: Garage connectivity Kit	OpenSprinkler: Automate your sprinklers	

## Appendix B

This appendix presents the hardware scheme of the notification device for promoting healthy eating behaviors.

As indicated in Section 3 the prototype developed to promote healthy eating behaviors has been equipped with a monochrome 0.96″ 128 × 64 OLED graphic display using the driver chip SSD1306 from Adafruit (Adafruit Industries Inc., New York City, NY, USA). The OLED display (U5 component in the schematic diagram) has been connected through a SPI 4-wire interface to the ESP8266-12E System on a Chip (U2 component). The ESP8266 also keeps an I2C two-wire serial communication with the tri-axial accelerometer + tri-axial gyroscope InvenSense MPU-6050 IMU (U4 component). These three components are supplied with a 3.3 V regulated DC voltage provided by the LF33ABDT-TR 3.3 V Low-Dropout regulator (STMicroelectronics Inc., Geneva, Switzerland) (U1 component). A low-cost TP4056 micro USB single-cell lithium battery charger with cut-off protection (NanJing Top Power ASIC Corp., Nanjing, Jiangsu, China) has been used to properly adjust the charging current supplied to the 3.7 V LiPo battery and to prevent its total discharge. The TP4056 is represented by the U3 component in the schematic diagram.

## Appendix C

This appendix presents the hardware scheme of the notification device for promoting physical activity in children. It is a simpler hardware scheme than the previous one from Appendix A. It keeps the LF33ABDT-TR 3.3 V LDO regulator, the ESP8266-12E and the TP4056 lithium battery charger which correspond to the components U1, U2 and U3, respectively. There are no OLED display and no IMU in this hardware scheme. Instead, a 5 RGB LED array (component U4) with integrated drivers from Adafruit is connected to the ESP8266-12E through the GPIO2 pin to provide visual cues. Lastly, this notification device also incorporates a feedback switch button (SW3 component) connected to the GPIO5 through a pull-up resistor. It is used to provide feedback to the system so that the notification has been attended by the end users.

## Appendix D

This appendix describes the questions asked to each evaluator and the scale used for each question.

1. Participant information
  - Name
  - Professional background
2. Prototype
  - What do you think is the main goal of the system? (open question)
  - How hard would it be to use the system? (0–10 scale)
  - How interested would you be in testing/using this system? (5pt Likert scale)
  - How satisfied are you with the look and feel of this system? (5pt Likert scale)
  - Would you buy this system? Why? (Yes/No and open question)
  - Do you have any thoughts on how to improve this system? (open question)
  - In three words, describe this system (open question)
  - Will this system be a good tool to promote healthy behaviors? (Yes/No and open question)
  - How likely is it that you would recommend this system to a friend/family member/patient? (0–10 scale)
3. Ergonomics of Human-System Interaction
  - For each category select a response (5pt discrete scale)
    - Perceived effectiveness
    - Perceived efficiency
    - Perceived satisfaction
    - Clarity
    - Discriminability
    - Conciseness
    - Consistency
    - Detectability
    - Legibility
    - Comprehensibility
    - Self-descriptiveness
    - Suitability for individualization
    - Suitability for learning
  - Is there anything else you would like to share? (open question)
